# Performance measurement and evaluation of health practitioner regulation: A scoping review protocol

**DOI:** 10.1371/journal.pone.0319507

**Published:** 2025-03-17

**Authors:** Patrick Chiu, Kathleen Leslie, Gina Jang, Tracey L. Adams, Natalie Thiessen, Janice Y. Kung

**Affiliations:** 1 Faculty of Nursing, University of Alberta, Edmonton, Alberta, Canada; 2 Faculty of Health Disciplines, Athabasca University, Athabasca, Alberta, Canada; 3 Department of Sociology, Western University, London, Ontario, Canada; 4 Geoffrey & Robyn Sperber Health Sciences Library, University of Alberta, Edmonton, AB, Canada; Xiamen University - Malaysia Campus: Xiamen University - Malaysia, MALAYSIA

## Abstract

Health practitioner regulation plays a fundamental role in public protection by overseeing and governing healthcare professionals to ensure they deliver safe health services. It also serves as a strategic lever to strengthen broader health system goals such as improving the accessibility of services, the sustainability of health workforces, and health system resilience. Although the goals of health practitioner regulation are easily articulated, achieving and evaluating these goals are far more challenging. Performance measurement and evaluation of professional regulators and regulatory systems are critical to improving regulatory processes and functions. This is especially important where there is rising government, public, and professional skepticism and mistrust of the effectiveness and efficiency of regulators across global jurisdictions. Although there is evidence that some health practitioner regulators and regulatory systems engage in performance measurement and evaluation, the similarities and differences remain unclear. The objective of this scoping review is to explore the nature, extent, and range of scholarship related to health practitioner regulatory performance measurement and evaluation. It will explore existing performance measurement and evaluation frameworks; the key principles and areas of focus of these frameworks; and the indicators, metrics and outcomes used to evaluate performance. The review will be conducted in accordance with the JBI guidelines for scoping reviews and will be reported following the Preferred Reporting Items for Systematic Reviews and Meta-Analyses Extension for Scoping Reviews. Database searches will include Ovid MEDLINE, Ovid EMBASE, CINAHL, Scopus, and Web of Science Core Collection. Gray literature will be identified through leading regulatory organizations, consortiums, and think tanks. Two independent reviewers will screen titles and abstracts followed by full-text and disagreements will be resolved by a third reviewer. Data will be analyzed using descriptive statistics and conventional content analysis. Results will be presented using evidence tables and a narrative summary.

Open Science Framework Registration: https://doi.org/10.17605/OSF.IO/WABTF

## Introduction

Professional regulation, in a broad sense, refers to legally defined rules governing entry into specific occupations and the conduct within them [1]. Fundamentally, regulation is intended to manage “the asymmetry in knowledge” [2, p. 595] between healthcare professionals and the general public, mitigate associated risks, and thereby promote public safety [3]. In the context of professional fields, regulation serves as a structured means to ensure that practitioners within a specific sector deliver their expertise safely, ethically, and in a manner that upholds public welfare [4]. Health practitioner regulation (HPR) specifically pertains to the oversight and governance of healthcare professionals to ensure they meet entry-to-practice criteria for education, competency, and ethical behaviour to practice safely [5]. HPR aims to protect the public through activities such as reviewing and accrediting education programs to ensure those entering the workforce are prepared to meet entry-level competencies; granting licensure to professionals who meet regulatory requirements; enforcing professional standards and ongoing professional development; and addressing complaints related to professional misconduct [3,4]. There is also growing recognition of the role that HPR plays as a strategic lever to advance broader health system goals, including improving access to care, managing the distribution of health services, promoting health system resilience, advancing the integration of new technologies, and supporting the sustainable development health workforces [2,6]. Others have also begun to explore the role and opportunities that HPR has in addressing society’s wicked problems such as worsening social inequities, climate change, and the rapid evolution of artificial intelligence [7].

The global landscape of HPR is characterized by its diversity across different jurisdictions, shaped significantly by each country’s legal traditions, political economy, and colonial history [2]. HPR systems vary widely in their legitimacy, scope, governance models, and operations [6]. The legitimacy of HPR systems can be generally categorized into statutory or non-statutory regulation. Their scope may be national or subnational and their governance may be led by governments, professions, independent authorities, oversight bodies, or meta-regulators [6]. The administration of these systems may also vary. For example, they may be managed by single agencies comprising boards that carry responsibility for different professions or functions, or individual profession or function-specific bodies [6]. Regardless, HPR is a crucial aspect of health system governance that balances risk prevention with the flexibility needed to address global and local health priorities and modern health challenges [6].

Studies emphasize the importance of regulatory bodies and systems being proactive rather than reactive, suggesting that regulatory approaches must evolve with healthcare innovations and societal shifts to remain relevant and effective in their protective role [8]. To adequately protect the public and address contemporary health challenges, HPR systems must be dynamic and adaptable [6]. This raises important questions about what it means to be an efficient, effective, and responsive regulator and regulatory system, and how regulatory performance is or should be evaluated.

Actors within health systems including governments, regulated professionals, and the public are showing growing skepticism in regulators’ ability to effectively protect the public. For example, several highly publicized professional conduct cases across jurisdictions have illustrated significant shortcomings of regulatory systems compromising public confidence [9–12]. The literature also suggests that many regulated professionals, irrespective of profession or jurisdiction, perceive their regulators as ineffective, bureaucratic, and costly institutions that they must navigate to avoid trouble [13]. Further, governments are increasingly becoming involved in traditionally self-regulated professions given growing concerns about their ability to protect the public. For example, regulatory reforms across global jurisdictions have seen the development of regulatory oversight bodies and greater government control over regulatory functions [14–16]. While there are strong examples of positive regulatory reform in some global jurisdictions, how the effectiveness and efficiency of health practitioner regulators, and regulatory arrangements as a whole, are evaluated remains unclear. HPR is inherently complex, and while the objectives of regulation are easy to articulate, delivering on and evaluating these objectives is far more challenging [17].

Globally, population health needs, the availability and accessibility of health services, and the nature of health workforces are evolving substantially. Greater attention is needed to assess whether regulatory philosophies, approaches, and systems are effectively addressing and mitigating emerging risks. The integration of new technologies and artificial intelligence is posing new social, legal, and technological threats to public safety [17–22]. Shifting demographics, emerging infectious diseases, and worsening non-communicable diseases are creating new population health challenges [6,17]. The demand for and the expectations of healthcare services are resulting in greater privatization and corporatization [6]. Generational differences in work-life balance are also creating new ways of engaging in professional work such as the shift towards self-employment [23]. All of these developments introduce new risks to the public and continue to shape the activities that regulated professionals are engaged in. As regulators grapple with increased government, public, and workforce pressures against the backdrop of shifting social, technological, economic, environmental, political, legal, and ethical developments, the need to evaluate their regulatory policies, practices, and systems to maintain and improve public trust and confidence becomes even more compelling.

A recent global integrative review of HPR systems identified that very few jurisdictions have implemented institutionalized arrangements for reviewing their regulatory systems to support continuous improvement [6,24]. The review found that many regulatory reforms have occurred as a result of ‘one-off’ reviews and inquiries into cases of regulatory failure or crises [24]. However, there is some evidence that regulatory performance reviews are being implemented more routinely and proactively in jurisdictions such as the United Kingdom, New Zealand, Australia and Canadian provinces such as Ontario. In the United Kingdom, the Professional Standards Authority for Health and Social Care conducts periodic reviews to evaluate regulatory performance using their Standards of Good Regulation [25]. Some regulatory bodies outside of the United Kingdom have implemented voluntary performance reviews using these standards [25]. New Zealand has also implemented legislated independent performance reviews for regulatory authorities [26]. Australia recently commenced a review of the National Registration and Accreditation Scheme to explore the governance, stewardship, responsiveness, and adaptability of their regulatory scheme and to identify ways to reduce unproductivity and unnecessary complexity [27]. In Ontario, Canada, the Ministry of Health has developed the College Performance Measurement Framework [28], whereby health practitioner regulators are required to report annually on a set of criteria. We are also aware that some individual regulators in the Canadian context have developed or are in the process of developing their organizational-specific key performance indicators [29].

Recent guidance on the design, reform, and implementation of HPR by the World Health Organization identified various considerations for strengthening regulatory systems and institutions. Monitoring and evaluation were identified as one way to reduce or minimize the regulatory practice gap, that is, when regulatory policies are not implemented in practice or when they do not meet the intended purpose or result in the desired outcome [6]. This can be a result of over or under regulation, or implementation of inappropriate regulatory policies and approaches. Routine or periodic monitoring and evaluation are critical to identifying the impact of regulation on patient, public, and health service delivery outcomes and assessing the efficiency and effectiveness of a regulatory system [6].

Despite the growing popularity of these evaluation frameworks in practice, we do not yet have a comprehensive understanding of the scope of HPR performance measurement and evaluation frameworks that exist; the contexts in which they are applied; and how the underlying principles, areas of focus, indicators and metrics, outcomes, and development processes compare. We conducted a preliminary search of MEDLINE, the Cochrane Database of Systematic Reviews, and JBI Evidence Synthesis, and no current or ongoing systematic reviews or scoping reviews on the topic were identified. As a result, summarizing the extant literature is a foundational step to inform whether existing regulatory performance and measurement frameworks can be spread and scaled across contexts, and where further development is needed.

The objective of this review is to identify the nature, extent, and range of scholarship related to health practitioner regulatory performance measurement and evaluation.

## Review questions

The questions guiding our review include:

1. What frameworks exist to evaluate the performance of health practitioner regulators?
2. What are the key principles underpinning health practitioner regulatory performance evaluation frameworks and what are the key areas of evaluation?
3. What indicators, metrics, and outcomes are used to evaluate regulatory performance?

### Inclusion criteria

#### Population.

Our population of interest includes health practitioner regulators. Health practitioners include all health professionals identified in the International Standard Classification of Occupations [30]. We refer to health practitioner regulators as authorities recognized by governments to govern the practice of health practitioners in the public’s interest.

#### Concept.

Our key concept of interest involves performance evaluation and measurement principles and frameworks. We define this as any efforts implemented or applied by regulators to assess their structures, processes, and outcomes. We draw on the adopted definitions provided by Leslie et al [24]. Structures refer to the social, technological, economic, political, legislative, ethical, equity, and demographic contexts. Processes refer to the functions and activities of regulators which may include internal governance and administrative processes and core regulatory functions such as setting entry to practice requirements, licensing qualified practitioners, maintaining a public register, administering and implementing continuing professional development programs, developing and enforcing professional standards, and addressing complaints and professional misconduct. Outcomes can be broadly conceptualized as the effectiveness and efficiency of a regulatory system in meeting patient, public, and health system goals.

#### Context.

Our review adopts a global approach and will focus on exploring regulatory performance measurement and evaluation within the context of regulatory systems established by governments or professional bodies under government delegation or recognition [24].

#### Types of sources.

We will consider all types of studies using quantitative, qualitative and mixed methods studies. However, given the review topic, we anticipate that the majority of relevant sources will not include empirical research. As a result, we will also include discussion papers, commentaries, and opinion papers if they include a substantive exploration of HPR and performance evaluation. Gray literature such as policy reports, legal reviews, and organizational reports will be identified through leading regulatory organizations. Systematic reviews will be excluded; however, reference lists will be screened for relevant studies. Books will be excluded. Unavailable sources will be excluded unless the full text can be accessed through interlibrary loan services.

## Methods

The scoping review will be conducted following the JBI methodology for scoping reviews [31]. This review was registered with the Open Science Framework on November 11^th^ 2024. We developed this protocol using the reporting guidelines in the Preferred Reporting Items for Systematic Reviews and Meta-Analyses Extension for Protocols (PRISMA-P) [32,33] as they apply to scoping reviews [33] (see S1 Appendix: PRISMA-P Checklist).

### Search strategy

Our search strategy will aim to locate both published and unpublished studies. With the support of a medical research librarian (JYK), we conducted an initial search of MEDLINE (Ovid) to identify sources on the topic (see S2 Appendix: Initial Search Strategy). The text words contained in the titles and abstracts of relevant sources, and the index terms used to describe the sources will be used to develop a full search strategy using the following databases: Ovid MEDLINE, Ovid EMBASE, CINAHL, Scopus and Web of Science Core Collection. The search strategy, including all identified keywords and controlled vocabulary terms (e.g., MeSH), will be adapted for each included database. We will screen the reference list of all included sources for additional sources. In addition to our database search, we will search the first 200 citations from Google Scholar and the websites of leading regulatory organizations such as the Professional Standards Authority for Health and Social Care, the International Association of Medical Regulatory Authorities, the National Council of State Boards of Nursing, the Australian Health Practitioner Regulation Agency, the Canadian Network of Agencies for Regulation, and the Council on Licensure, Enforcement, and Regulation. Additional organizations may be identified during the screening process. We will use the following keywords including: performance evaluation, performance measurement, and regulatory performance. We will also consult subject matter experts in the field to identify sources that may not be identified in our databases and gray literature search. Non-English sources will be excluded due to the lack of translation services. Given the increased focus on performance evaluation and measurement over the last decade, we will limit the search to literature published after 2013.

### Study/source of evidence selection

We will collate and upload all identified citations into Covidence (2023), a review management software program [34]. Following a pilot test using 10% of the papers, two independent reviewers will screen the titles and abstracts to assess them against the inclusion criteria. Potentially relevant sources will be retrieved in full text. The full text of selected citations will be assessed against the inclusion criteria by two or more independent reviewers. We will record and report reasons for excluding sources during full-text review that do not meet the inclusion criteria. Conflicts between the reviewers at each stage of the selection process will be resolved through consensus meetings and with a third reviewer. Citations of included sources will be uploaded into Zotero, a reference manager software programme [35]. The reporting of the results will be guided by the Preferred Reporting Items for Systematic Reviews and Meta-Analyses Extension for Scoping Reviews (PRISMA-ScR) [36].

### Data extraction

Data will be extracted from sources included in the scoping review by two or more independent reviewers using a data extraction tool developed by the reviewers (see S3 Appendix: Data Extraction Tool). The extraction tool will include details about the population (health profession regulators), concept (performance measurement and evaluation) and context (all geographic regions). We will pilot the extraction tool using 10% of the included sources and refine the tool as needed. Disagreements that arise between the reviewers will be resolved through discussion, or with an additional reviewer/s. The extraction tool may be further modified and revised as needed during the review process. Any modifications made to the extraction tool will be detailed in the scoping review. If appropriate, authors of sources will be contacted to request missing or additional data, where required. Critical appraisal of individual sources will not be completed as this is generally not required for scoping reviews.

### Data analysis and presentation

Results will be organized using evidence tables to ensure data are presented in a clear and structured format. Descriptive statistics will be used to illustrate frequency counts and percentages for data such as the year of publication, evaluation principles, evaluation areas of focus, metrics or indicators, and performance evaluation outcomes. We will use conventional content analysis [37] to analyze the extracted data as it will allow for the development of broader categories based on coded data. We will accompany the descriptive statistics with a narrative summary to ensure the evidence presented responds to the review objectives and questions. To ensure the overall reporting quality of this scoping review, reporting will follow the PRISMA-ScR [36]. Using an adapted PRISMA flow diagram, all retrieved records from the search strategy, included and excluded records from primary and secondary screening, and records retrieved from other methods will be reported to enhance the reproducibility of this scoping review.

## Discussion

This scoping review will address a gap in the literature by describing and summarizing the current landscape of health practitioner regulatory performance measurement and evaluation. Specifically, it can broaden our understanding of the breadth and depth of evaluation frameworks including the areas of focus; the metrics and indicators used; the extent to which regulatory performance is linked to professional, patient, and population health outcomes [2]; and by extension, ideas of what it means to be a high-performing health practitioner regulatory system. Enhancing regulatory performance and stewardship is critical to creating modern regulatory architecture [38].

Existing literature highlights the importance of evaluation research to identify regulatory structures and processes that lead to the highest impact [24]. This literature also suggests that using standardized frameworks to evaluate various regulatory structures and arrangements can support stronger comparisons across jurisdictions [24]. However, to enable this, scholars and regulatory leaders first require a comprehensive understanding of existing performance measurement and evaluation frameworks. Our findings have the potential to inform regulatory reforms in several ways. First, a global review can help identify whether there are frameworks that health practitioner regulators commonly use across professions and jurisdictions. Second, our review can illustrate the frameworks applied to individual regulatory bodies versus those that apply more broadly to a HPR system to enhance conceptual clarity. Third, exploring the underpinning principles that guide the development of performance and measurement frameworks can uncover the evolving beliefs, values, and philosophies within the field of HPR. Fourth, identifying the evaluation and measurement areas of focus, indicators and metrics, and evaluation outcomes can guide the standardization of evaluation metrics that can be applied across professions and jurisdictions, promoting consistency and comparability in assessing regulatory performance. Lastly, many HPR systems struggle with data collection and highlighting commonly used metrics can inform regulators on what data to collect to inform improvements. This, in turn, can enhance regulators’ capacity to demonstrate their impact on influencing professional behaviour, patient safety, and health system outcomes.

Since the purpose of the review is to provide an overview of regulatory performance evaluation frameworks, we will not be assessing the quality of the literature or the effectiveness of the evaluation frameworks. With the inclusion of English-only language sources, we may miss relevant non-English language publications. Additionally, given the dynamic context of regulatory reform and the fast-moving nature of this field, and we may not capture all relevant gray literature. We anticipate some heterogeneity in the design, focus, and application of HPR performance measurement and evaluation frameworks across jurisdictions and professions. However, the focus of our review is to map the literature on the topic (i.e., scoping review) rather than to synthesize the best available evidence (i.e., systematic review). We will carefully contextualize our findings to facilitate our interpretation of the data. Despite these limitations, this scoping review will address a timely and practical issue around how to evaluate and improve the efficiency and effectiveness of HPR and will offer a comprehensive synthesis across different health professions and jurisdictions.

## Supporting information

S1 Appendix PRISMA-P checklist. (PDF)

S2 Appendix Initial search. (PDF)

S3 Appendix Data extraction tool. (PDF)
