# Supplementary material for: Performance measurement and evaluation of health practitioner regulation: A scoping review protocol
Source: PLoS One. 2025 Mar 17;20(3):e0319507. doi: 10.1371/journal.pone.0319507 (PMC11913261; doi:10.1371/journal.pone.0319507)
Supplement: S2 Appendix — (PDF) [file pone.0319507.s002.pdf]

S2 Appendix: Initial Search Strategy

Database	Search Strategy
**MEDLINE**<br><br>Ovid MEDLINE(R) ALL 1946 to October 9, 2024	1. ((acupuncturist* or allergist* or anatomist* or an?esthesiologist* or anesthetist* or audiologist* or cardiologist* or chiropractor* or clinician* or dentist* or dermatologist* or diabetologist* or dietician* or doctor* or doula or doulas or endocrinologist* or gastroenterologist* or general practitioner* or geriatrician* or gynecologist* or h?ematologist* or hospitalist* or immunologist* or intensivist* or internist* or medical resident* or midwife or midwives or neonatologist* or nephrologist* or neurologist* or neurosurgeon* or nurse or nurses or nutritionist* or obstetrician* or oncologist* or ophthalmologist* or optometrist* or osteopath or osteopaths or otolaryngologist* or pathologist* or p?ediatrician* or pharmacist* or pharmacologist* or phlebotomist* or physician* or podiatrist* or prosthetist* or psychologist* or psychiatrist* or pulmonologist* or radiographer* or radiologist* or radiotherapist* or rheumatologist* or surgeon* or therapist* or toxicologist* or urologist* or veterinarian*) adj4 (registration* or regulat* or licensing or licensure or certificat* or credential*)).ti,ab.<br>2. exp *Licensure/<br>3. ((worker* or workforce or personnel or practitioner* or provider* or professional* or employee* or staff* or navigator*) adj4 (registration* or regulatory or regulation* or licensing or licensure or certificat* or credential*)).ti,ab,kf.<br>4. ((licens* or regulat*) adj1 (authorit* or agency or agencies or body or bodies or college* or council* or board*)).mp.<br>5. or/1-4<br>6. ((performance or transparenc* or risk* or safety or framework or efficien* or effective*) adj4 (evaluat* or measur*)).ti,kf. or ((performance or transparenc* or risk* or safety or framework or efficien* or effective*) adj4 (evaluat* or measur*)).ab. /freq=2<br>7. regulat* impact assessment*.mp.<br>8. (good adj2 regulat*).mp.<br>9. professional self-regulation*.mp.<br>10. right-touch regulation.mp.<br>11. (regulat* adj3 stewardship).mp.<br>12. or/6-11<br>13. 5 and 12<br>14. animals/ not (animals/ and humans/)<br>15. (veterinary or rabbit or rabbits or animal or animals or mouse or mice or rodent or rodents or rat or rats or murine or hamster* or pig or pigs or piglets or swine or porcine or horse* or equine or cow or cows or cattle or bovine or goat or goats or sheep or lambs or ovine or monkey or monkeys or trout or marmoset$1 or canine or dog or dogs or feline or cat or cats or zebrafish).ti.<br>16. 14 or 15<br>17. 13 not 16

	18. limit 17 to (english language and yr="2013 -Current")
