# Supplementary material for: Performance measurement and evaluation of health practitioner regulation: A scoping review protocol
Source: PLoS One. 2025 Mar 17;20(3):e0319507. doi: 10.1371/journal.pone.0319507 (PMC11913261; doi:10.1371/journal.pone.0319507)
Supplement: S3 Appendix — (PDF) [file pone.0319507.s003.pdf]

S3 Appendix: Data Extraction Tool

Data Fields	Details
ID	
Author	
Year	
Jurisdiction	Identify the jurisdiction where the regulatory performance evaluation and measurement framework was developed.
Health profession(s)	Identify whether the tool was developed for a specific health profession or if it can be applied to health professions regulation in general.
Regulator-specific versus regulatory system	Identify if the framework was developed to evaluate the performance of a specific regulatory body or an entire regulatory system.
Underpinning principles	Identify the principles underpinning the regulatory performance and measurement framework.
Evaluation and measurement areas of focus	Identify the core areas of evaluation and measurement.
Indicators and metrics	Identify any indicators or metrics used to enable performance evaluation and measurement.
Outcomes	Identify any outcomes of performance evaluation and measurement within the framework.
Process of development	Identify any processes used to develop the performance evaluation and measurement framework.
